# A Bibliometric Study on the Social Validity of Telepractice in Autism Spectrum Disorder

**DOI:** 10.3390/ijerph20010419

**Published:** 2022-12-27

**Authors:** Rómulo J. González-García, Gabriel Martínez-Rico, Claudia Escorcia-Mora, Pau García-Grau

**Affiliations:** 1 Doctoral School, Catholic University of Valencia San Vicente Martyr, 46001 Valencia, Spain; 2 Campus Capacitas, Catholic University of Valencia San Vicente Martyr, 46001 Valencia, Spain

**Keywords:** social validity, telepractice, autism spectrum disorder, bibliometric

## Abstract

The field of telepractice is generating increasing interest in recent years. In recent years, early childhood services have used resources such as support for interventions in families with children with autism spectrum disorder. Due to this situation, the social validity of such resources has emerged in this sector, receiving individual attention from academics and practitioners. However, a further deepening of such analyses is needed. Therefore, the main objective of this work is to analyze papers published in the Web of Science on social validity and telepractice in autism spectrum disorders. Bibliometric analysis allows us to discover the current state of a research field, to identify the main authors, articles, and topics, and to propose future lines of research to develop it further. Articles published between 2000 and 2021 were analyzed quantitatively, and by co-occurrence of words and authors. Subsequently, through bibliographic linking, the articles were grouped into different clusters. Five central themes were found, with social skills and the effectiveness of intervention programs being the most developed areas of research. Furthermore, studies focusing on evidence-based practices are necessary for the development of this research field. Thus, the analysis of social validity in the field of telepractice in children with autism spectrum disorders is a developing field within the early childhood sector.

## 1. Introduction

Children with ASD often present with social communication deficits that may hinder their interaction and communication with others and their participation in activities of daily living. These deficits are key criteria in the diagnosis of the disorder [1]. In addition, another characteristic of children with ASD is restricted and repetitive interests or behaviors, which also negatively affect both overall performance as well as the establishment of social relationships [1,2]. On the other hand, behavioral problems such as aggression are common in children with ASD and can be a barrier to promoting participation and full inclusion [3].

According to the World Health Organization (WHO), it is estimated that 1 in 100 children have an Autism Spectrum Disorder (ASD) [4]. The Autism and Developmental Disabilities Monitoring Network (ADDM) conducted monitoring in 11 communities across the U.S. in 2018 [5]. The results of the study show that 1 in 44 8-year-olds (2.3%) have ASD. It was also observed that the chances of identifying ASD were 4-fold higher in boys than in girls. There was no significant difference in race (black, white, Hispanic, Asian, or native) in the Pacific Islands. However, in several places, the percentage of Hispanic children in whom ASD was identified was lower compared to black or white children. The results indicate that the number of children identified with autism is higher than in previous reports. The reasons that seem to explain the current prevalence of ASD are: (a) advances in diagnostic procedures, (b) broadening of diagnostic criteria, (c) recommendations for universal screening for ASD, (d) increased public awareness of the disorder, (e) increased public awareness of ASD, (f) increased awareness of ASD, (g) increased awareness of ASD, and (h) increased awareness of ASD. These findings indicate that there are many children with ASD who need services and support now and will need them during their development into adolescence and adulthood.

In Spain, a study developed by the association Autismo España (Autism Spain) in collaboration with the General Directorate of Disability Policies, a prevalence of approximately 1 case per 100 births was calculated; that is, 1% of the population could have ASD [6]. This would imply that in Spain, there could be more than 450,000 people with ASD. Among the conclusions of this study, it was also revealed how since 2015, there has been a significant increase in the population with identified ASD. It documents that a reliable diagnosis during infancy and childhood, and that the earlier that intervention is initiated, the greater the likelihood of an improved developmental trajectory. It is argued that early intervention is more cost and time efficient than a “wait and see” approach [7].

This reality should force administrations to become aware of the impact of the lack of resources to provide supports for people with ASD and the need to establish public policies that respond to the needs and demands of this group and their families [8]. While findings from both the U.S. and Spain show that much progress has been made to overcome disparities in the identification of ASD in children, more work is needed to ensure early evaluation and diagnosis of children as early as possible, from the moment developmental problems are detected [7]. Likewise, it is necessary to advance in research and carry out both an evaluation and an intervention adjusted to scientific evidence, which enables children and their families to advance in their communication and social interaction.

### 1.1. Telepractice Applied to ASD

In 2020, the crisis caused by COVID-19 showed the situation of extreme vulnerability of people with disabilities and especially people with ASD. During the lockdowns decreed by governments around the world ASD, people saw their rights of movement limited and their routines were altered by having to stop going to their centers and services [9]. As a group with support needs, it was difficult for people with ASD to understand the reason for isolation, understand hygiene and social distancing measures, and therefore regulate their behavior. This situation increased the risk of challenging behaviors, which negatively affected the emotional wellbeing and quality of life of the entire family household [10,11,12], and forced families, in most cases, to entirely assume the supports that these people needed.

Against this background, support services had to adapt to the new reality, and telepractice proved to be an effective tool to provide support to this group and their families [13]. However, telepractice is not new. It is directly related to the origin of telemedicine, and this, in turn, is connected with the advance of telecommunications. The first use of telemedicine to transmit video, images, and complex medical data occurred in the late 1950s. In 1959, the University of Nebraska used interactive telemedicine to transmit neurological exams. Additionally, in the 1960s–1970s, NASA (National Aeronautics and Space Administration) incorporated it, since it needed to keep track of astronauts traveling to space. This technology was originally developed to connect patients living in remote areas to doctors working in urban areas [14].

Telepractice is defined as the use of telecommunication technologies as a means to provide remote care services [15] and allows the combination of real-time (synchronous) telehealth technologies, as well as storage and transmission (asynchronous) [16]. This method has been effective and has allowed transdisciplinary collaboration among support services professionals to be maintained [17]. Until the pandemic, telepractice was a known resource, but very common in early intervention [18].

It is currently used not only in medical fields, but also in psychology, education, and to provide social services, under the name of telepractice or teleintervention [8]. This is characterized by the use of computer applications and videoconferencing to transmit instructions and connect caregivers (parents, educators, therapists) with specialists who are remote. In the autism field, we find that it is used as a way to provide support, training and implementation of effective practices, as well as evaluation and intervention with families [19].

There are many programs that are carried out through telepractice especially for providing behavioral support [20], allowing the incorporation of communication systems [21], improving communication and socialization skills, and coaching parents, which have proven successful both in the implementation and in the generalization of the skills worked [8,19,22,23].

The use of teleintervention as a widely used support procedure in the provision of early intervention services during the time of pandemic has also been studied. Although it was an unusual procedure, remote intervention, using a virtual methodology, has taken center stage. The results reflect a positive impact of the family-centered paradigm (FCP) to generate, through teleintervention, a greater family participation in early intervention. Likewise, this approach is positively related to the satisfaction of the family needs and expectations [24].

### 1.2. The Social Validity of Interventions

All scientific knowledge has as its fundamental objective to represent reality faithfully, so its procedures must be as refined as possible, without contaminations that cloud that strength. Thus, a series of techniques, processes, rules, and procedures have been developed to achieve results of excellence and quality, which certify that social research has been conceived and executed with the necessary rigor to define its main results as a science [25]. The analysis of the social validity of the intervention in early childhood requires evaluating the perception and satisfaction of parents or primary caregivers. This involves analyzing the variables of the implementation of each program itself, evaluating its effectiveness and fidelity [26].

In this context, research developed by [27] analyzed the effect of the use of technology in the training of caregivers who used the methodology based on the token economy for its application during non-preferred routine activities. The results showed that caregivers acquired the necessary skills for their use and a greater increase in positive interactions with children. In this study, measures of social validity that served to assess caregivers’ acceptance of the use of the token economy were also taken into account. These results were applied to the design of intervention programs for people with ASD.

In a literature review, Stavropoulos et al. [28] examined the reliability, social validity, and feasibility of using telepractice when diagnosing people with ASD. The conclusions of their study suggest that the information collected through telepractice has an accuracy and reliability between 80 and 91%, a sensitivity between 75 and 100%, and a specificity between 68.5 and 100%, compared to the diagnosis made in person. Therefore, the social validity of telepractice in diagnosis turned out to be acceptable. Concerning the advantages, the researchers found that specialists appreciate the flexibility that comes from being able to analyze videos with the family or be able to review them at other times. They also highlight the possibility of seeing the child in his natural environment and cost savings, as well as the high degree of satisfaction with the evaluation procedures carried out remotely [29,30,31].

On the other hand, in relation to parent training procedures, studies have been carried out such as that of [22] in which digital technology was used to train parents in video analysis as a means to increase self-instruction practices and generalization to other objectives. The results showed that the instruction was effective, the parents changed the practices, generalized to other objectives and these maintained over time. Regarding social validity, parents found the intervention acceptable and feasible [29].

In the same line, in the study presented by [19], the objective was to evaluate to what extent parents of children with autism can implement a set of newly acquired strategies and analyze the impact they had on the communication skills of their children. At the end of the study, parents learned to use the communication strategies implemented through the Internet and their children were satisfied with the communication implemented by the parents. Similarly, their children’s interactions and communication initiation increased and remained for the most part after the completion of the study, thus concluding that distance entertainment and training can be a feasible and effective method of service delivery.

When it comes to contextualizing and evaluating the scientific production of a specific area, bibliometric analyses seem to be an effective tool. This study technique has been used in various branches of knowledge [32,33,34,35] so its usefulness as a tool for exploratory analysis of a specific topic is evident. That is why the use of bibliometric analysis allows the use of bibliographic indicators to analyze the specific literature of a given field of research [36,37].

For all the above, this study aims to analyze through a bibliometric analysis the scientific field concerning ASD, telepractice, and social validity. The social validity variable, in this study, refers to the social acceptability of the intervention in the context of ASD. This search is interesting since this terminology has undergone a substantial change in recent years, coinciding with the greater accessibility to platforms that allow better access to remote viewing. For this reason, greater attention has been put in the research field regarding the subject. Therefore, in view of the increasing interest, this bibliometric analysis aims to respond to the current state of this research field. For this purpose, the design of the study has been oriented through the following research questions:

RQ1—What is the evolution of the articles published on autism spectrum disorders and their relationship with telepractice and social validity over time?

RQ2—Which authors have published the largest number of articles related to autism spectrum disorder and its relationship with telepractice and social validity? Which have been the most cited?

RQ3—Which countries, academic journals and institutions have published the most on the topic of autism spectrum disorders and their relationship with telepractice and social validity and what is the impact factor of these journals?

RQ4—What co-authorship networks, cooperation networks between countries, co-citations and co-words study the relationship between autism spectrum disorder, telepractice and social validity?

RQ5—What are the main themes studied in this field of research?

The structure of this work is organized, first, presenting the methodology, specifying the different bibliometric methodological techniques and the software used to obtain the results. These results will then be presented through the different tables and bibliometric maps. After that, the results will be discussed and finally a series of conclusions will be presented, the future implications presented by the study, as well as the limitations of said work.

## 2. Materials and Methods

### 2.1. Data Collection

This work analyzed all articles published and indexed in the Web of Science Core Collection™ (SSCI, SCI-Expanded) on autism spectrum disorder and its relationship with telepractice and social validity. In this work, only the Web of Science (WoS) database has been considered, since in this database the scientific journals with the greatest impact, and therefore, the scientific articles with the greatest scope within the research field are considered [38].

For this work, an advanced search has been carried out by selecting the topic field. This search field performs its search both in title, abstract or keywords and is also the most widely used in most bibliographic studies [37,39,40]. Thus, the search equation used was: (TS = (autism spectrum disorder OR ASD OR autism)) AND TS = ((“social validity”) OR (“telemedicine” AND “telepractice” AND “telehealth”)).

We conducted and compared this search on 6 October 2022. It is crucial to present the date of collection of documents because the database is constantly updated [41].

**Figure 1:**
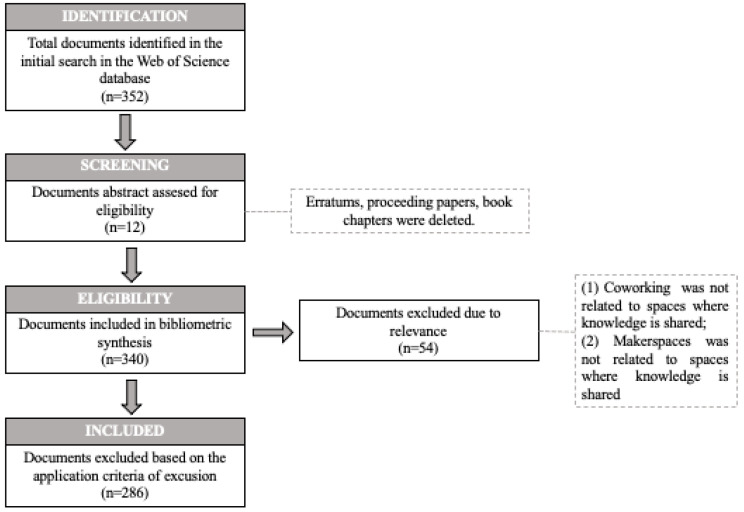
PRISMA flow diagram.

The study was limited to research articles in the strict sense, including only original papers and reviews. Therefore, documents such as: editorials, book reviews, conference abstracts, letters, editorials and news and bibliographic articles were excluded. The search string was also limited to 2021, although there was no language restriction. The initial search retrieved 352 documents through 2021. All these articles were then reviewed to select the final articles. This information can be seen in Figure 1. This procedure was followed to discard those articles that did not refer to the topic under study (eligibility). The authors adopted the PRISMA (Preferred Reporting Items for Systematic Reviews and Meta-Analyses) framework [42,43,44] to review and select articles for literature search. 

After this procedure, 286 documents remained in the final review database. Finally, these selected articles were downloaded in plain text with data on the year of publication, authors, author affiliation, title, abstract, journal, subject area, references, and number of citations.

### 2.2. Bibliometric Analysis

After having the data in plain text, duplicate and unrecognized records were identified and homogenized. Thus, the total number of articles was reviewed to avoid duplication and errors and to find missing data in some registries (institutions, countries, and year of publication). Subsequently, the analysis was carried out in two different stages.

First, HistCite (version 10.12, University of Pennsylvania, Philadelphia, PA, USA) was used to sort the data collected by authors, years, countries, journals, and references cited. This software presents the information in an orderly and detailed manner. Thus, with this software, the basic bibliometric analyses were carried out: the number of articles per year, the number of articles per author, the number of articles per journal and the number of articles per country. However, Hitscite not only displays quantitative indicators, but also quality indicators: Total Global Citation Score (TGCS) and TLGCS (Local Global Citation Scores). Therefore, this work takes into account both quantitative and quality indicators. The Global Citation Score (GCS) refers to the total number of citations received by the articles selected in the analysis conducted through WOS. The Total Local Citation Score (LCS), on the other hand, represents the number of citations received in the WOS database only by the articles selected in the analysis.

Next, the R studio v.3.4.1 software was used through the R bibliometrix package http://www.bibliometrix.org (accessed on 30 October 2022) [33] to prepare the data for analysis of co-authoring networks. Likewise, collaboration networks among countries, co-keywords and thematic analyses were carried out using the same software. The data was imported into R Studio and converted into a bibliographic data framework. Bibliometrix covers the entire workflow, while the other software only implements a part of it [45]. Thus, this software was used to analyze the basic information of the search chain performed, the index of collaboration among countries, the map of collaborations among countries, the word cloud of the authors and the analysis of the strategic diagrams. Strategic diagrams based on joint word analysis make it possible to identify the main research topics and emerging research topics in this specific field of research. In addition, increasing number of studies are investigating the development of emerging research fields in order to detect relevant topics to delimit research areas through the use of different techniques, such as bibliographic linking, co-word analysis or historiographic analysis [32,46]. Thus, thirdly, the analysis of co-word networks and bibliographic linkage and thematic analysis were used. Keyword co-occurrence was used to analyze the most prevalent and emerging topics related to ASD, social validity, and telepractice. A bibliographic linkage analysis was also performed to identify the different clusters. Bibliographic linkage measures the similarity between two articles by identifying the number of references they have in common. In addition, the number of references cited in the articles does not change over time. Therefore, this analysis, compared to the co-occurrence analysis, is not influenced by the timing of its performance [47]. For this reason, it is advantageous to use it for systematic reviews of the literature [37]. For the correct interpretation of these two network maps (co-word maps and bibliographic coupling maps), it is necessary to consider that each cluster is related to a color.

## 3. Results

The search carried out has reported a total of 286 articles in a total of 76 journals. This is how a total of 959 authors from 25 different countries are reported. The average number of citations per document is 19.58. Regarding keywords, a total of 723 keywords and 732 author keywords have been found. Finally, according to the number of authors per document is around 2.92, with a collaboration index of 3.02. This information can be seen in Table 1.

### 3.1. Basic Indicators

This first results section presents the basic indicators. Thus, the evolution of the works and citations by year, the number of works and citations by author, by institution and by country, and the journals are presented. Finally, the evolution of the keywords of the authors according to the years of publication.

#### 3.1.1. Years

**Figure 2:**
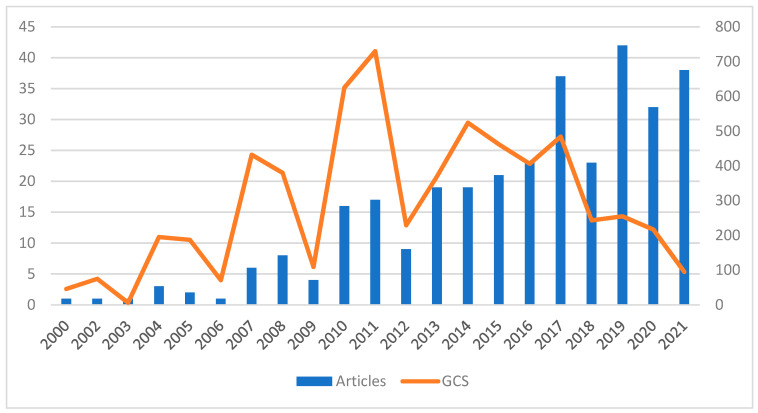
Evolution of the number of articles and reviews published over the years and the number of global citations (2000–2021). GCS = Global Citation Score.

The number of articles published on this topic has increased over the years although there are a total of five records prior to the year 2000. It is in the last 20 years, however, where there has been a considerable increase in the number of publications on this ASD, telepractice or social validity. A turning point is observed from 2013 (19), and 2021 stands out as the year with the highest number of articles published (38). According to the number of citations, articles written in 2010 (625) and 2011 (730) have had the highest number of citations. Figure 2 shows its evolution:

#### 3.1.2. Authors

A total of 959 researchers have published at least one article on ASD, telepractice or social validity. However, the researchers with the highest number of publications are Meadan (14) and Machalicek (11). Subsequently, authors such as Barton, Blair, Carter or Lang have published a total of seven articles. The results are shown in Table 2.

On the other hand, the authors who have received the highest number of citations in their research are Lang (GCS = 461) and O’Reilly (GCS = 430). Next, Sigafoos (GCS = 366), Lancioni (GCS = 359) and Machalicek (GCS = 275) have more than 250 citations in the total number of their publications related to the subject. However, as far as the authors’ h-index is concerned, Lancioni stands out with an h-index of 50, beginning his publications in 1980. More detailed information on these researchers can be found in Table 3.

#### 3.1.3. Institutions

As shown in Table 4, both Anadolu University and Vanderbilt University have published more than 30 papers related to ASD, telepractice or social validity. However, the University of Texas Austin has a higher overall citation/post ratio, with a score of 33.50. It should also be noted, among the universities with the highest number of publications, the universities of North Carolina, University of Oregon and Vanderbilt University have coefficients greater than 10 points.

#### 3.1.4. Countries

**Figure 3:**
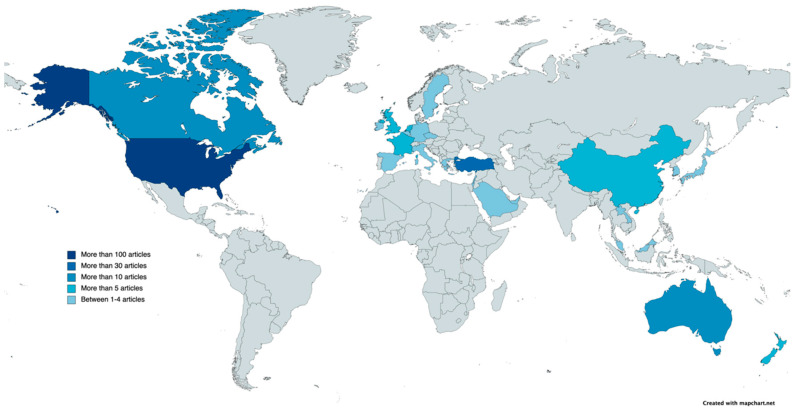
Number of articles published by country.

Researchers from a total of 25 countries have published at least one article on this research topic. The country that has published most articles is the United States (196), followed by Turkey (33), Australia (17) and Canada (14). Among the other countries, researchers from New Zealand, China and the United Kingdom have published more than 5 articles. All other countries have published 4 articles or less. Secondly, in terms of the countries that have received the highest number of citations throughout the WOS, the United States stands out in first place (GCS = 3450), followed by Australia (GCS = 453), and in third place Turkey (GCS = 281). This information can be seen in the Figure 3.

#### 3.1.5. Journals

A total of 83 journals have published at least one article on this topic (see Table 5). From all of them, there are a total of 10 journals that have published more than ten articles on the subject. Thus, the journal with the highest number of articles published is “Research in Autism Spectrum Disorders” with 30 published articles, being also the journal with the highest number of global citations (GCS = 672). Next, the journals “Journal of Autism and Developmental Disorders” and “Journal of Applied Behavior Analysis” have published more than 25 articles.

On the other hand, according to the impact factor of the journals that have published the largest number of articles, the journal “Journal of Autism and Developmental Disorders” is the one with the highest impact factor (JCR = 4.345), followed in second position by the “Research in Autism Spectrum Disorders” (JCR = 3.293) and in third position by the “Journal of Applied Behavior Analysis” (JCR = 2.809). The results can be seen in Table 5.

### 3.2. Co-Citation Analysis

Next, the analysis of co-citations will be presented. This section will first introduce co-authoring networks, followed by cross-country collaboration networks and finally keyword networks. All this will be presented through its corresponding figure.

#### 3.2.1. Co-Authorship Networks

**Figure 4:**
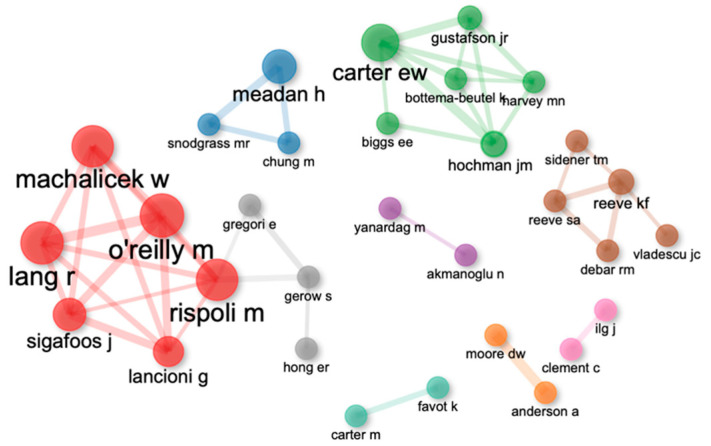
Co-authorship networks.

The 9 co-authorship networks among the 31 principal investigators who have published joint articles on this topic are presented. In addition, for each of the networks a cluster is presented using Louvain’s algorithm. Specifically, there is a network of six researchers whose most prominent authors are Machalicek, O’Reilly and Rispoli. This network presents a relationship with another network of three authors whose most outstanding author. Additionally, you can see another network of six researchers whose most prominent author is Carter and another network of five authors will focus on Reeve. The rest of the networks are composed of three authors or fewer. Figure 4 shows the different collaboration networks:

#### 3.2.2. Collaborations between Countries

**Figure 5:**
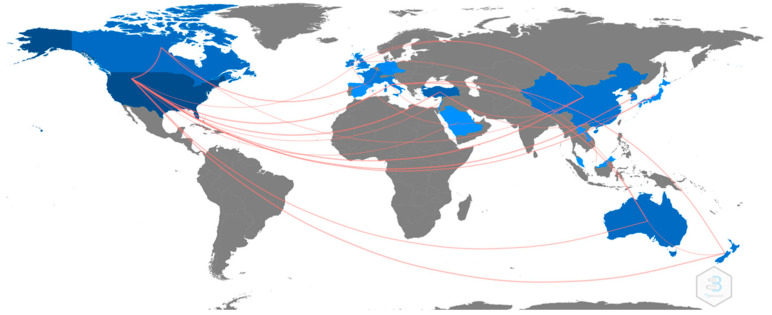
Country Collaboration Networks.

In terms of collaborations between countries, as shown in Figure 5, shows the collaboration networks between countries. As can be seen, these are composed of various countries around the world. The United States stands out as the most collaborative country, followed by Australia. It is observed that the United States collaborates with several countries, although collaboration networks between countries are skyrocketing globally. Figure 5 shows collaboration between countries.

#### 3.2.3. Co-Word Analysis

**Figure 6:**
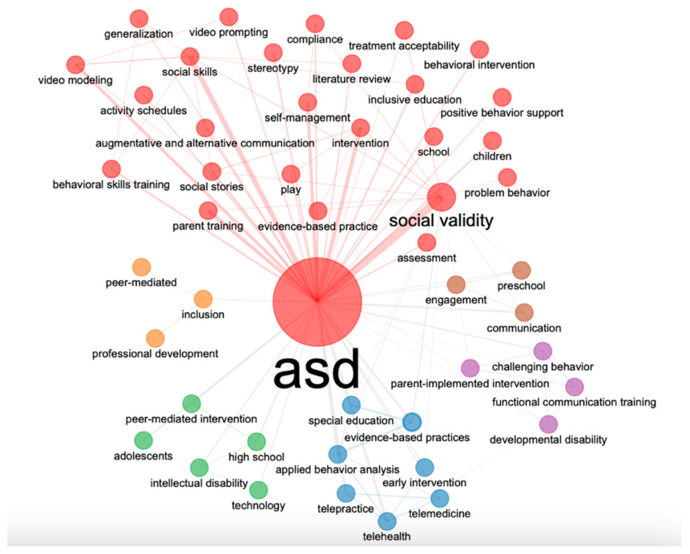
Co-word networks.

Five main groups of keywords were found. The first is composed of 27 words (red cluster), which refers to aspects related to the autism spectrum, behaviors and interventions focusing on social validity and social skills as the most recurrent connections. The second network is composed of seven words (blue cluster), which refers to issues related to special education and evidence-based practices. The third network consists of five words (green cluster), in which terms oriented towards more advanced stages of the individual such as adolescents or high school are observed. Next, a fourth network composed of four words (violet) that focuses mainly on aspects such as communication or the implementation of interventions. Finally, two additional networks were found composed of three words focusing on inclusion and professional development (orange cluster) and terms such as engagement, preschool, and communication (brown cluster). Figure 6 shows the different clusters.

### 3.3. Thematic Analysis

Finally, this third section presents the results of the thematic analysis. First, the bibliographic coupling analyses are presented, and then, a strategic diagram of the different topics obtained in the search. All these results are represented by figures.

#### 3.3.1. Bibliographic Coupling

**Figure 7:**
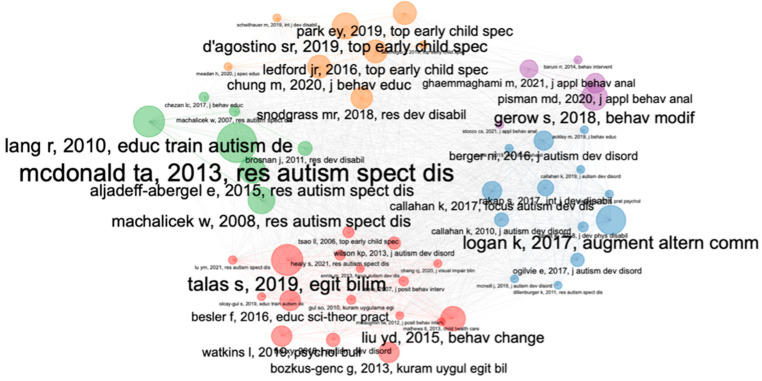
Bibliographic coupling analysis.

A bibliographic coupling analysis was performed. Only documents that were connected to each other were selected, limiting the analysis to 50 documents. In this way, the results were distributed in five different clusters (one color per cluster). These clusters can be seen in Figure 7.

Red cluster: Social skills

This group is the largest and consists of 17 documents. The theme of these works is related to the analysis of social skills in the field of autism spectrum disorder. Within this cluster, we can highlight works such as that of [48] that analyze whether the multiple or unique presentations of the presentation of social stories in the teaching of the behaviors of individuals with ASD differ in terms of effectiveness and efficiency. On the other hand, articles such as that of [49] evaluate the effects of a self-management intervention partially implemented by parents that incorporated a video-model for discrimination training in the improvement of social skills in a child with ASD. This intervention was effective in improving this participant’s social skills. Likewise, documents such as those of [50] highlight the effectiveness of video modeling as a resource when it comes to acquiring skills in children with ASD. Specifically, this work shows how mothers of children with autism were able to prepare video recordings and implement video modeling with full treatment integrity, such video modeling being effective to teach a game skill to children.

Blue Cluster: Social Validity

This group consists of 13 documents. The theme of these works is related to the analysis of the social validity of the various interventions. Featured articles in this cluster, such as that of [51] present a systematic review on alternative and augmentative communication systems to support children with autism spectrum disorder. On the other hand, articles such as that of [52] also analyze through a review of the literature by which the social validity of evidence-based practices in children with ASD is analyzed. In his search, the authors focus on the analysis of seven categories associated with social validity. Likewise, documents such as those of [53] evaluate tools that allow measuring the social validity of interventions in the development of skills of children with ASD.

Orange cluster: telepractice

This cluster is composed of a total of eight articles. The theme is related to telepractice and its relationship with the social validity of ASD interventions. Works such as the one carried out by [54] carried out a systematic review to evaluate the characteristics, objectives, procedures and results of assessment practices on social validation in which researchers trained professionals to implement naturalistic behavioral interventions in children with ASD. Additionally, works such as that of [55] are included within this grouping, where a series of single-case studies are systematically reviewed where they try to establish the prevalence of social validity and evaluate the scientific rigor of these. For their part, studies such as that of [56] sought to determine the extent to which the social and ecological validity of interventions designed to increase the social skills of children with ASD was demonstrated.

Green cluster: Effectiveness of intervention programs

This group is composed of seven articles. The theme of these works is related to the analysis of the effectiveness of various interventions. Works such as that of [57] which reviews the literature stands out. This paper examined the participation of teachers in intervention research for children with ASD from 1996 to February 2008. Another review by [58] also analyzes the publications that have applied intervention programs in adolescents with ASD, noting that the interventions have focused their attention on seven areas called: (a) social skills; (b) communication skills; (c) challenging behavior; (d) academic skills; (e) vocational skills; (f) independence and self-care; and (g) physical development. On the other hand, studies such as that of [59] reviewed the effectiveness of these intervention programs, focusing on five areas of attention: (a) academic skills, (b) communication skills, (c) functional life skills, (d) play, and (e) social skills.

Violet cluster: Functional training

This cluster is composed of a total of five articles. The theme is related to the training carried out for the improvement of various functions of children with ASD. Works by [60] address a systematic review on functional communication training implemented by parents of children with ASD. Other studies such as that of [61] analyze the various treatments based on the use of teaching techniques related to an alternative form of communication to reduce the problematic behavior of children with ASD. For its part, the paper in [62] tries to evaluate the appropriate procedures to train caregivers of children with ASD through the application of a combination of strategies designed to establish the relationship and language skills, keeping play as the preferred context.

#### 3.3.2. Strategic Thematic Analysis

**Figure 8:**
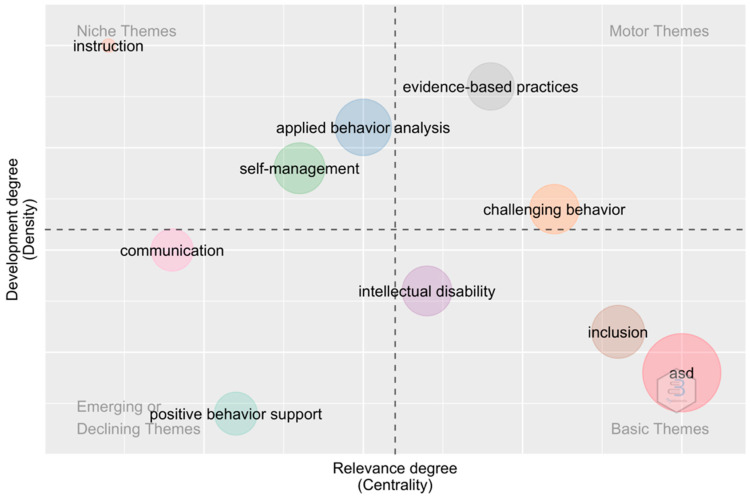
Strategic diagram.

Finally, the strategy diagram for the theme in question is presented. The size of the spheres represents the number of occurrences of these keywords (see Figure 8). The upper right quadrant corresponds to motor topics, which means that they are developed and important for the field of research. The upper left quadrant refers to highly specialized topics, with well-developed internal links but unimportant external links meaning they have limited importance to the field of study. The lower right quadrant corresponds to the basic and cross-cutting themes, which means that these topics are important for the theme and refer to general themes which are cross-cutting to the different areas of research. Finally, the terms that appear in the lower left quadrant are known as emerging or declining themes, meaning they are underdeveloped or marginal.

The results of this analysis report that, from the upper right quadrant, we find terms such as “evidence-based practices” and “challenging behavior”. Regarding the topics of the upper left quadrant, such as “applied behavior analysis”, “self-management” and “instruction”, they have developed internal links, but external links that are not very relevant and, therefore, only have a certain specific importance.

The topics in the lower left quadrant, in this case, are terms such as “communication” and “positive behavior support”. Finally, the topics of the lower right quadrant appear in basic cross-sectional and general terms such as “ASD”, “intellectual disability” and “inclusion”.

## 4. Discussion

The results of this study demonstrate that the field of autism spectrum disorder continues to attract the attention of researchers from different parts of the world. This interest is due to the importance they have acquired in the field of autism spectrum disorder, issues such as the social validity of interventions, the use of telepractice or the verification of effectiveness in programs [19]. Evidence of this is the existence of consolidated co-authoring networks, whose researchers stand out for the large number of articles published. In addition, these collaborations are global, occurring in multiple institutions around the world. Therefore, from the relevant studies in this field, it is crucial to study the development and incidence of ASD from a global perspective, to obtain a better understanding, and in turn promote the optimal development of all the agents involved in this question.

Among the topics explored, studies conducted before and during the pandemic have focused on the effectiveness of telemental diagnosis and intervention in ASD, user satisfaction with its use, benefits and limitations of telepractice in ASD [63]. An interesting research gap, which has been further explored in recent manuscripts, has been the evaluation and comparison of clinicians’ and users’ views on this type of telemental services for ASD [64]. Given the spread of telehealth care services during the pandemic, it was essential that researchers also explore the expectations and concerns of users and providers about telepractice for ASD, in order to create a model of telehealth care that meets their needs as far as possible [63,64].

Hence, resources related to ASD must be strengthened in relation to social validity optimally [65,66]. It is essential that the professionals in charge of the care of this community meet the needs and achieve higher levels of person-oriented adequacy, involving the entire environment of the child [67,68]. On the other hand, the field of telepractice is an important line of research in development, and its analysis and particular characteristics must be deepened [68]. Although the number of publications on this topic has increased in recent times, they focus on the analysis of the effectiveness of interventions on skill development. Therefore, more research is still needed on the social validity of the resources used. This fact agrees with the statement of [53], who point out that the field of social validity should continue to develop due to the variability of resources available for supporting people with ASD and their families.

The results show how, mainly since 2015, there has been a clear increase in publications related to autism spectrum disorder, social validity and telepractice. However, studies such as that of [28] indicate that they must continue to deepen the field, given the variability and particularity of the cases.

Thus, a large number of authors have been interested in the study of autism spectrum disorder and telepractice or its social validity. This research reports a total of 959 authors. It is worth noting the great variability that exists in relation to the number of journals that have published documents in this sector. This shows how, the growing interest in the subject receives wide acceptance and has a future projection within the field of research. At this level we find how various institutions, mainly from the United States, have been participants in the development of this theme, being their universities the ones that have developed the greatest scientific production. It also stands out, as in Latin American and African countries there are hardly any records, so it is essential to pay attention to these territories when developing work related to autism spectrum disorder.

In relation to collaborative networks, these focus on authors from North American institutions, where most of the co-authoring networks that carry out work in the field of research can be observed. Additionally, it is worth mentioning the presence of authors from the University of Anadolu, due to the large number of publications made.

With regard to the thematic axes, the importance of social validity in the field of autism spectrum disorder is highlighted [52]. Several authors have developed tools to ensure the validity of the resources used in interventions with children. Guaranteeing and measuring these interventions have been one of the most popular axes in terms of publications, as is the case of the study presented by [28].

Additionally, aspects such as the effectiveness of intervention programs, such as the implementation of communication systems [21,69], reading habits and behavior management [70,71] or training programs to support people with ASD or training parents on specific learning strategies such as the studies of [20,22,27,72] have also been frequent in this field. Thus, it is recommended to pay attention and deepen its study in order to develop evidence-based practices [52].

### Limitations

The present study has been based exclusively on conducting a search in the Web of Science database, so it would be interesting to extend this search and analysis to other databases in order to deepen the publications on the subject. Additionally, it is possible that some articles related to the topic have been omitted from the search carried out, which may directly limit the results of this research, as other bibliometric studies have also pointed out [73]. This situation could be due to words that have been indexed in WoS by authors and publishers. However, we believe that this is very unlikely and, in any case, it would be very scarce and would not affect the meaning and globality of the results found and described in this study. Likewise, it is necessary in this type of study to define the social validity variable operationally to rigorously delimit the object of study of this analysis.

## 5. Conclusions

Within the field of autism spectrum disorder, both social validity and telepractice prove to be very relevant areas in the coming years. With the rise of new technologies and given the quantity and variability of resources that occur in the sector, it is necessary to clarify what practices allow optimizing resources in this field. This article presents an overview of the evolution and current state of this field of research. It shows how analyzing social validity is essential when applying the different resources that professionals have in the interventions carried out in the field of ASD, especially those developed through a methodology based on telepractice. Additionally, it is worth mentioning how measuring the effectiveness of these interventions, as well as training programs for professionals and family members are the most prominent fields of study.

In recent years, the network of support services has been incorporating telepractice as an intervention modality in the development of its intervention programs [74]. The methodology based on telepractice can guarantee better access and continuity of services and, consequently, increase the satisfaction and commitment of the caregiver with the programs, obtaining, in turn, positive results for the child [66,75]. Telepractice has the potential to improve the quality of the service delivered [16], suggesting that in the coming years, remote intervention will become a main, or at least complementary, resource in the provision of Early Childhood Intervention services [75,76]. The conclusions of this research may be useful for researchers on the autism field to expand their knowledge in the application of telepractice as an intervention methodology, considering as a constant reference the social validity of the intervention.

## Figures and Tables

**Table 1:** Main information about data.

Main Information about Data	
Journals	76
Articles	286
Average years from publication	6.3
Average citations per documents	19.58
Average citations per year per document	2.30
References	9043
*Document contents*	
Keywords plus (ID)	723
Author’s keywords (DE)	732
*Authors*	
Authors	959
Authors of single-authored documents	16
Authors of multi-authored documents	943
Authors collaborations	
*Countries*	25
Single-author documents	16
Documents per author	0.34
Authors per document	2.92
Co-authors per documents	3.89
Collaboration index	3.02

**Table 2:** Authors with the highest number of publications.

Author	Nb	h-Index	PY	Institution	Lcs	Gcs	GCS/Nb
Meadan H	14	17	2000	University of Illinois	38	129	9.21
Machalicek W	11	26	2007	University of Oregon	29	275	25
Barton EE	7	26	2007	Vanderbilt University	21	198	28.28
Blair KSC	7	12	2007	University of South Florida	13	72	10.28
Carter EW	7	42	2001	Varnderbilt University	22	247	35.28
Lang R	7	37	2007	Texas State University	36	400	57.14
DeBar RM	6	8	2005	Caldwell University	4	42	7
Douglas SN	6	10	2013	Michigan State University	3	34	5.66
Tekin-Iftar E	6	14	2002	Anadolu University	6	86	14.33
959 authors		-	-	-	-	-	-

Note: Nb-number of articles; PY—year of the first article published; LCS—Local Citation Score; GCS—Global Citation Score.

**Table 3:** Authors with the highest number of citations.

Author	GCS	Institution	h-Index	PY
Lang R	461	Texas State University	37	2007
O’Reilly M	430	University of Texas at Austin	44	1991
Sigafoos J	366	Victoria University Wellington	48	1986
Lancioni G	359	Universita degli Studi di Bari Aldo Moro	50	1980
Machalicek W	275	University of Oregon	26	2007

Note: PY—year of the first article published; GCS—Global Citation Score.

**Table 4:** Publications by institutions.

Institution	Country	Nb	LCS	GCS	GCS/Nb
Anadolu University	TUR	33	25	291	8.81
Vanderbilt University	USA	35	39	380	10.85
University of Illinois	USA	19	42	155	8.15
University of Texas Austin	USA	16	48	536	33.50
University North Carolina	USA	14	11	340	24.28
University of Oregon	USA	14	26	179	12.78
356 institutions	-		-	-	-

Note: Nb—number of articles; LCS—Local Citation Score; GCS—Global Citation Score; TUR—Turkey; USA—United States of America.

**Table 5:** Journals by the number of publications and citations received (LCS and GCS) and Impact Factor (JCR).

Journal	Nb	LCS	GCS	GCS/Nb	JCR (2021)
Research in Autism Spectrum Disorders	30	44	672	22.40	3.293
Journal of Autism and Developmental Disorders	27	52	629	23.30	4.345
Journal of Applied Behavior Analysis	26	54	648	24.92	2.809
Journal of Behavioral Education	24	10	142	5.92	2.469
Topics in Early Childhood Special Education	20	47	563	28.15	2.313
Education and training in autism and developmental disabilities	19	6	123	6.47	1.078
Journal of developmental and physical disabilities	18	23	153	8.50	1.517
Behavioral Interventions	17	7	109	6.41	1.269
Journal of Positive Behavior Interventions	14	32	260	18.57	2.597
Focus on Autism and Other Developmental Disabilities	12	27	254	21.17	2.434
83 journals		-	-	-	-

Note: Nb—number of articles; LCS—Local Citation Score; GCS—Global Citation Score.

## Data Availability

Not applicable.
